# First-in-Human Phase 1 Studies in Oncology: The New Challenge for Investigative Sites

**DOI:** 10.5041/RMMJ.10074

**Published:** 2012-04-30

**Authors:** Marc Salzberg

**Affiliations:** Medpace Inc., Cincinnati, OH, USA and Università degli Studi, Milan, Italy

**Keywords:** Challenges, oncology, phase 1, study design

## Abstract

Phase 1 first-in-human studies with anti-cancer products differ from other phase 1 studies in that they are evaluated in patients rather than healthy volunteers. The rationale design of targeted drugs triggers changes in the design of these studies. Patient populations are more precisely defined and pose a challenge to the efficient inclusion of study patients. Objectives shift from the definition of a maximum tolerated dose to the evaluation of a recommended phase 2 dose. Other challenges related to the efficacy and safety profile of novel targeted anti-cancer drugs call for changes in designing first-in-human studies, such as definitions of biological doses, collection of fresh tumor tissue for surrogate marker analyses, and the management of infusion-related reactions with monoclonal antibodies.

Consequently, the conduct of phase 1 clinical trials in oncology requires changes. Corresponding education with particular focus on phase 1 trials and on the complex drug development process needs to be an integrated part of the medical oncology curriculum for physicians and nursing staff. This is a crucial element for institutions to remain or become clinical research sites for phase 1 studies, and to participate in the drug development process of novel anti-cancer compounds in the future.

According to the Annual Report of the Pharmaceutical Research and Manufacturers of America (PhRMA), nearly 900 medicines and vaccines are in development to fight cancer.^1^ Phase 1 first-in-human (FIH) studies with anti-cancer products differ from other phase 1 studies in that they are evaluated in patients rather than in healthy volunteers. The safety profile of anti-cancer products does not allow for testing in healthy volunteers, and investigational compounds are often a welcomed treatment option in the absence of effective alternatives for cancer patients.

In the last century, predominantly cytotoxic chemotherapies have been developed. The objective of phase 1 trials with those compounds was to administer the highest doses possible in order to determine the maximum tolerated dose (MTD). The rationale design of products targeting the downstream signaling process in the replication of cancer cells triggers changes in the design of FIH studies. A major difference is that patient populations are more precisely defined. In addition, objectives shift from the definition of an MTD to the evaluation of a recommended phase 2 dose (RP2D), since targeted therapies and even chemotherapeutic agents do not necessarily require the highest possible dose to be efficacious for target modulation and clinical activity.^2^

For example, chemotherapeutic agents have been shown to inhibit or retard the growth of tumor blood vessels at low doses, but with frequent and prolonged administrations. This metronomic chemotherapy is typically associated with fewer toxicities and allows for an efficient inhibition of the target; thus, this may be a better approach for FIH studies.^3^ The optimal biological dose defines the threshold at which that product is efficacious, but not yet toxic. The challenge is to avoid under-dosing patients, but at the same time to maintain reasonable dose escalation steps. Data from preclinical research and improved study designs help to overcome this hurdle in phase 1 studies. Simon and colleagues developed the accelerated titration design, which aims at making phase 1 studies more efficient and reduces the number of patients required. The distinguishing features of this design include a rapid initial escalation phase, intra-patient dose escalation, and the ability to analyze trial results using a dose-toxicity model that incorporates parameters for inter- and intra-patient variation in toxicity and cumulative toxicity.^4^ However, the risk of missing important toxicity and pharmacokinetic information may be higher with accelerated titrations, which should be considered when designing a phase 1 study protocol. This design might be more suitable for late-stage phase 1 studies conducted in patient populations more likely to benefit from the investigational product.

The development of a monoclonal antibody also poses challenges with regard to its administration. Infusion-related reactions (IRRs) are a common side-effect of antibodies that can lead to interruption and termination of the therapy and can even result in fatalities in extreme cases. The implementation of prophylactic measurements such as H1- and H2-blockers, steroids, and paracetamol or acetaminophen and the prolongation of the infusion might help to alleviate the incidence and severity of IRRs, but any implementation of such measures in phase 1 trials influences the further development of the compound substantially.^5^ Vast experience is required to carefully manage the prevention and treatment of such IRRs.

Another challenge in the conduct of scientifically sound phase 1 trials is the analysis of surrogate markers from tumor tissue. The collection of fresh tissue often requires study-specific biopsies. Paraffin-embedded tumor blocks are easier to obtain, although pathology institutions not involved in the clinical study are frequently reluctant to provide such samples for reasons related to their standard operating procedures or data protection laws. Every effort should be made to obtain such material, if its analysis can provide useful information concerning the definition of patient populations suitable for treatment with the investigational product and for the evaluation of the RP2D in the absence of an MTD.^6^

This trend towards personalized medicine in which tumor tissue from each patient is precisely defined might reduce the importance of the histology. The future testing of a combination of targeted molecules as opposed to classical cytotoxic agents creates a paradigm shift in the definition of the phase 1 patient population in oncology. While a rather heterogeneous cancer population was included in phase 1 trials in the past, the twenty-first century calls for rather precisely defined cancer patients with very specific tumor types. This approach was first used with receptors such as estrogen, progesterone, HER2, or EGFR,^7^ for which tumor tissue is stained for the expression of various proteins in parallel. There is clear evidence that triple-negative breast cancer patients have a different prognosis and require a different therapeutic approach than hormone receptor-positive and/or HER-positive tumors.^8^ Also, the qualitative definition of targets influences treatment approaches. For example, kras-mutant colorectal cancer is resistant to treatment with the EGFR antibody cetuximab, but kras-wild-type tumor tissue responds rather well to the treatment with this antibody.^9^ The restriction of phase 1 trials to subpopulations makes these studies more costly, since the requirement for highly specific patient populations triggers a longer recruitment period and a higher number of participating study sites. However, the overall drug development process should benefit in the long term. Higher response rates lead to a reduced number of patients needed for phase 2 and 3 studies and, thus, a reduced duration of the overall clinical development process for successful drug candidates.

In addition to the discussed changes in clinical research and drug development, the legal requirements for the conduct of clinical trials have changed substantially in the past decade and add to the complexity of clinical studies today.^10^

Consequently, we need to rethink the conduct of phase 1 clinical trials in oncology. The inclusion of subpopulations as described above limits the number of qualifying patients per site. Hence, such studies need to be conducted as multi-institutional projects in order to be completed in an efficient and timely manner. The involvement of more than three study sites, however, should be discouraged, since each investigator may only manage a limited number of patients, which dilutes valuable individual experience.^11^ Thus, multiple factors need to be considered when determining the number of required study sites. The feasibility process should include a discussion concerning the balance of time-lines and quality. Access to the patient population suitable for the study and ambitious but realistic time-lines both usually require a higher number of sites, whereas the co-ordination of treatment slots and communication between the investigative sites would be best achieved with a lower number of study centers.

Protocol compliance requires a sophisticated organization with experienced and dedicated investigators who can manage the requirements for the collection and adequate preparation of tumor tissue, the molecular and genetic staining of such material, pharmacokinetic sampling at various time points (sometimes well beyond regular working hours), and last but not least the management of patients enrolled in such studies. The fact that such trials are conducted at several centers requires regular communication between sites, the sponsor, and other parties involved. Updates in terms of safety profiles, including potential adjustments to the use of the investigational product during the study, patient slot allocation, and other operational aspects need to be reviewed and discussed on an on-going basis.

Thus, the minimum requirement to qualify as a phase 1 clinical research center is the availability of an experienced principal investigator, who is typically supported by a dedicated sub-investigator. A study co-ordinator ensures that patients are scheduled for the visits according to the study protocol and all necessary evaluations are performed. Such tests are carried out with the support of research or study nurses, who are often also involved in the administration of the investigational product. Storage and preparation of the investigational product is performed in collaboration with the hospital pharmacy. Other departments need to be involved in the conduct of FIH studies as well, including surgery for tissue collection and referral of patients, pathology for analyses, a laboratory for routine and specialty testing of liquids, and others depending on the nature of the studied disease. In addition, the hospital administration is involved for legal aspects of study contracts with the sponsor and eventually for budgeting purposes.

Hospitals interested in participating in the clinical research and development process of new molecules need to ensure that educated staff and infrastructure are available for the management of the complex process of phase 1 studies, but also for phase 2 and 3 clinical trials. Often start-up funding through grants and the institution itself is needed before a phase 1 clinical research center has a balanced budget through revenue generated from the conduct of studies sponsored by the pharmaceutical industry, by co-operative study groups, or other sponsors. Once such a unit is established and functional, the added value to the quality of the patients’ management becomes a key success factor in the reputation of institutions and staff participating in FIH studies. Academic sites and tertiary hospitals with access to large patient populations typically see the highest number of patients suffering from advanced cancer who have exhausted the standard treatment options. Phase 1 studies are often the last hope for those patients. Hence, institutions which intend to provide treatment options for such patients will be obliged to follow the above-mentioned requirements for modern drug development in order to qualify for participation in FIH studies in oncology. Education in the conduct of clinical studies in oncology with particular focus on phase 1 trials and on the complex drug development process needs to be an integrated part of the medical oncology curriculum for physicians and nursing staff. This is a crucial element for institutions to remain or become clinical research sites for FIH studies in oncology.

The pharmaceutical industry invests a great deal of time and money educating its associates in drug development. It is of paramount importance that investigators, their research teams, and the investigative institutions be similarly educated in the nuances of developing anti-cancer products if they aim to take part in the drug development process of novel anti-cancer compounds in the future.

## Abbreviations:

FIH: first-in-human;
IRRs: infusion-related reactions;
MTD: maximum tolerated dose;
PhRMA: Pharmaceutical Research and Manufacturers of America;
RP2D: recommended phase 2 dose.
